# Supplementary material for: Small Angle X-ray Scattering Sensing Membrane Composition: The Role of Sphingolipids in Membrane-Amyloid β-Peptide Interaction
Source: Biology (Basel). 2021 Dec 25;11(1):26. doi: 10.3390/biology11010026 (PMC8772848; doi:10.3390/biology11010026)
Supplement: Supplementary file 1 [file biology-11-00026-s001.zip › biology-1472726-supplementary.pdf]

Small angle X-ray scattering sensing membrane composition: the role of sphingolipids on membrane-amyloid β-peptide interaction

Rita Carrotta[1*], Maria Rosalia Mangione[1], Fabio Librizzi[1] and Oscar Moran[2]

[1] CNR, Istituto di Biofisica - Via Ugo La Malfa 153 - 90146 Palermo, Italy

[2] CNR, Istituto di Biofisica - Via De Marini, 6 - 16149 Genova, Italy

*Corresponding author

SUPPLEMENTARY MATERIAL

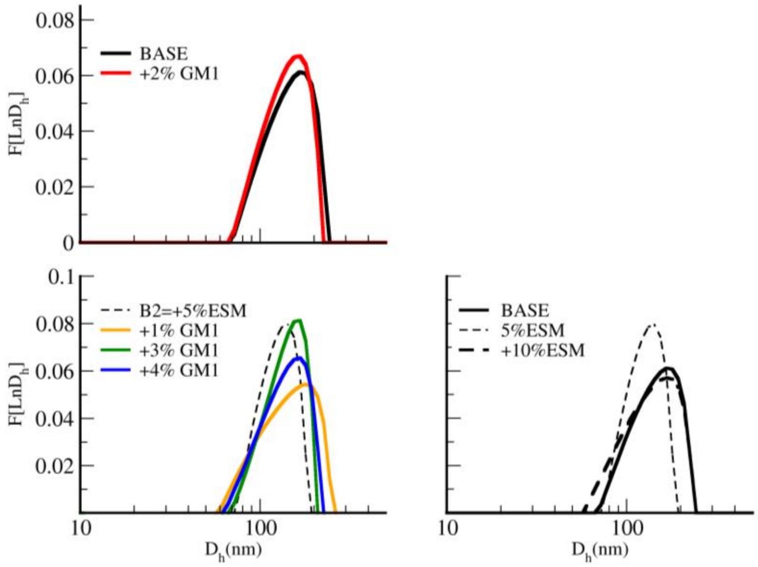

Figure S1. Dynamic light scattering size distributions for the various LUV samples used. The size distributions as a function of the hydrodynamic diameter which is related to *Γ*, that is to the decay of the intensity autocorrelation function, corresponding to a single species. The two variables are related according to the expression D_h = K*T* $q^2/(3\pi\eta\Gamma)$, where K is the Boltzmann constant, *T* the temperature, *q* the wave vector corresponding to the scattering angle *θ* (90° in our case), with $q = (4\pi/\lambda)\ \sin\theta/2$. Details on the method to obtain the size distribution from dynamic light scattering data can be found elsewhere [21].

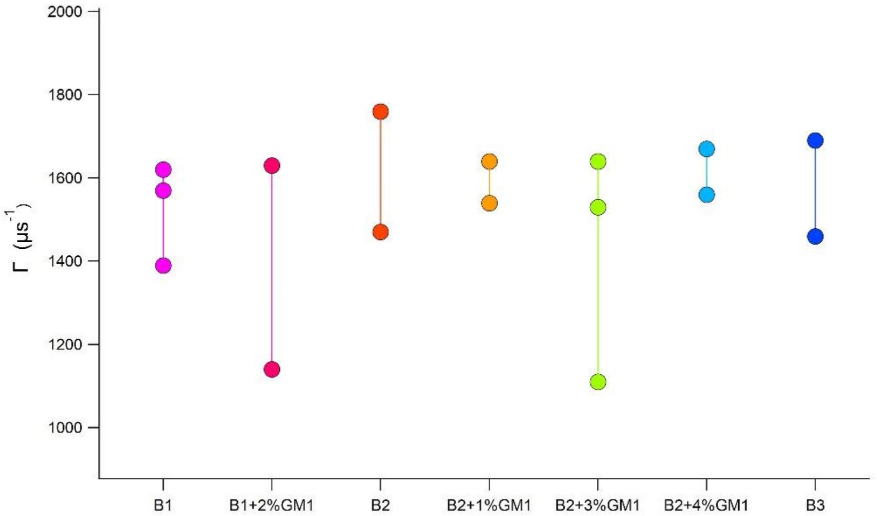

Figure S2. Decay constant *Γ*, calculated from the distribution obtained by the CONTIN-like analysis (of the intensity autocorrelation function. Data in the graphic are the individual measurements for each bilayer matrix (two to three replicates per each experimental condition).

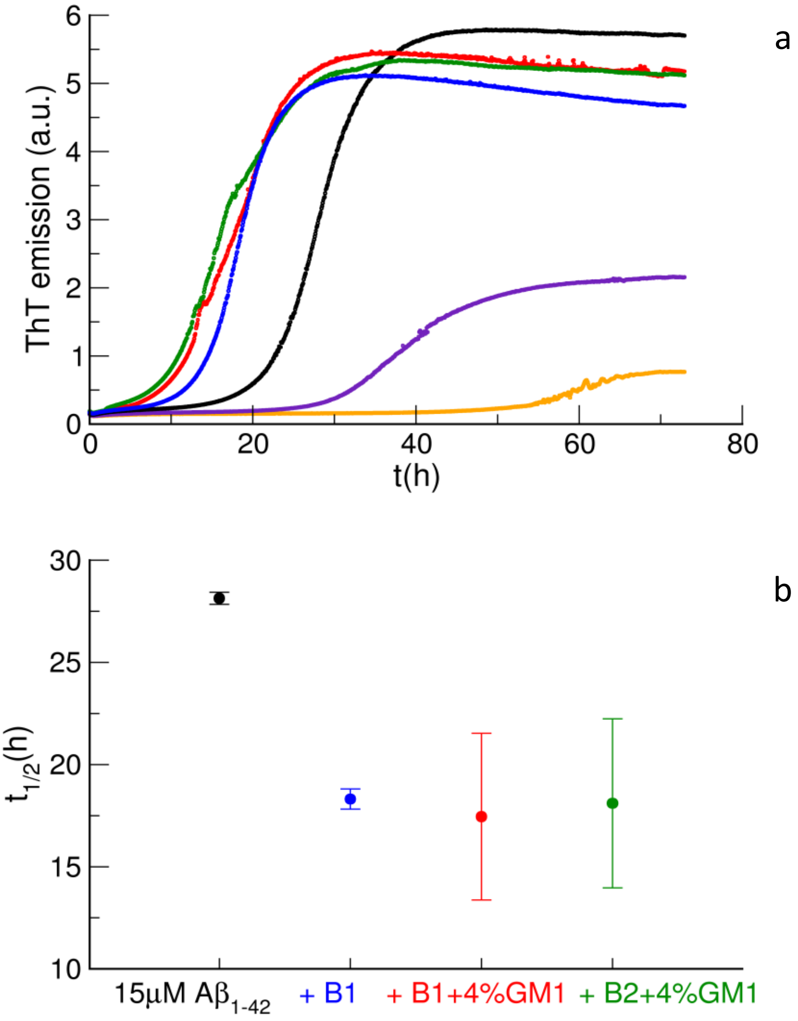

Figure S3: Kinetics of $A\beta_{1-42}$ alone at different concentration: 4.75µM (orange), 7.5 µM (violet) and 15µM (black) and at 15µM in the presence of different LUVs, matrix B1 (blue), B1+ 4%GM1 (red) and B2+ 4%GM1 (**a**). Half growth time of the ThT aggregation kinetics for the different samples. Error bar is calculated as standard deviation from three replicates (**b**). The ratio lipid:$A\beta_{1-42}$ was 250:1.
